# Listen to your heart: a critical analysis of popular cardiology podcasts

**DOI:** 10.3389/fmed.2024.1278449

**Published:** 2024-07-15

**Authors:** Harish Kamalanathan, Lewis Hains, Stephen Bacchi, Wrivu N. Martin, Ammar Zaka, Flynn Slattery, Joshua G. Kovoor, Aashray K. Gupta, Peter Psaltis, Pramesh Kovoor

**Affiliations:** ^1^Department of Cardiology, Gold Coast University Hospital, Southport, QLD, Australia; ^2^Department of Research, School of Medicine, University of Adelaide, Adelaide, SA, Australia; ^3^College of Medicine and Public Health, Flinders University, Adelaide, SA, Australia; ^4^Department of Research, Royal Adelaide Hospital, Adelaide, SA, Australia; ^5^College of Health, Medicine and Wellbeing, The University of Newcastle, Callaghan, NSW, Australia; ^6^Department of Cardiology, Westmead Hospital, Westmead, NSW, Australia

**Keywords:** cardiology, medical education, cardiology podcasts, medical education podcasts, cardiology learning

## Abstract

**Purpose:**

Podcasts are an increasingly popular medium for medical education in the field of cardiology. However, evidence suggests that the quality of the information presented can be variable. The aim of our study was to assess the quality of the most popular cardiology podcasts on existing podcast streaming services, using tools designed to grade online medical education.

**Results:**

We analyzed the five most recent episodes from 28 different popular cardiology podcasts as of 20th of September, 2022 using the validated rMETRIQ and JAMA scoring tools. The median podcast length was 20 min and most episodes were hosted by professors, subspecialty discussants or consultant physicians (87.14%). Although most episodes had only essential content (85%), only a small proportion of episodes provided detailed references (12.9%), explicitly identified conflicts of interest (30.7%), described a review process (13.6%), or provided a robust discussion of the podcast's content (13.6%). We observed no consistent relationship between episode length, seniority of host or seniority of guest speaker with rMETRIQ or JAMA scores.

**Conclusions:**

Cardiology podcasts are a valuable remote learning tool for clinicians. However, the reliability, relevance, and transparency of information provided on cardiology podcasts varies widely. Streamlined standards for evaluation are needed to improve podcast quality.

## Introduction

Podcasts are downloadable or streamable audio files that have become a popular medium for medical education (1–4). The convenience and ease through which information can be accessed have made podcasts an increasingly influential means of disseminating medical information. Accordingly, several prominent cardiology journals, cardiac societies, industry sponsors and universities regularly publish podcast episodes, with some podcasts reaching up to 84,000 episode-downloads per month (5).

Information accessed via podcasts can change the skillset and practice of listening clinicians (6). However, there are reports of variation in the quality of medical education podcasts by expert consensus (7, 8), and although several validated tools exist to assess medical education quality, there is currently no validated means by which to evaluate podcast quality. Without rigorous peer review, the translation of information obtained from podcasts into clinical practice may not be evidence-based.

The aim of our study was to assess the quality of the most popular cardiology podcasts on existing podcast streaming services, using tools designed to grade online medical education. Our *a priori* hypothesis was that the podcasts would score highly (>75%) among the rating scales, with podcasts from professional bodies being more likely to have a higher score.

## Methods

### Search method

We searched for cardiology podcasts, using the search term “cardiology” on September 30 2022, on the podcast directories, *Apple Podcasts* and *Spotify*. *Apple Podcasts* and *Spotify* were chosen as they are the world's two most used podcast platforms, accounting for 65.3% of all total listeners, with the next most used being the Web Browser which accounts for 3.5% of total listeners (9).

Inclusion criteria were any podcast with a title or author that explicitly mentioned “cardiology,” “heart,” or “cardiac.” Exclusion criteria were: (1) the podcast had not released an episode within the last 2 years, (2) the podcast had < 5 episodes, (3) the podcast was in video format, or (4) the podcast was not in English.

A consecutive sampling technique was used for both podcast directories. Podcast shows were screened in the sequence they appeared in the search results for “cardiology” on each respective platform, as of September 30 2022. The top 20 podcasts in each directory that met the inclusion criteria and demonstrated no exclusion criteria were compiled, then duplicate podcasts shows were removed. The five most recent episodes published up until September 30 2022 from the included podcast shows were retrieved and independently assessed by two authors.

### Scoring tools

We assessed the podcasts using two validated scoring tools: The rMETRIQ Score and the Journal of American Medical Association (JAMA) core quality standards. The rMETRIQ score is a 7 part questionnaire that assesses an online resource with questions grouped into three broad domains: the content quality of delivery, credibility and review processes (7, 8, 10–12). Each question can receive a score between 0 and 3 and the tool specifies clearly demarcated requirements to achieve each score, with a total possible score of 21.

The JAMA Benchmark Criteria is a streamlined assessment of online medical information and requires publications to meet four fundamental standards: authorship, attribution, disclosure and currency. These criteria are precisely defined in the 1997 paper by Silberg et al. (13). The total JAMA benchmark score was determined by awarding 1 point for each criterion that was present, allowing a minimum score of zero and maximum of four points.

The aformentioned evaluation metrics (rMETRIQ and JAMA) are designed to objectively assess resources independent of subject matter expertise and thus did not require previous cardiology knowledge (10–13).

### Adaptation of scoring tools to podcast format

The above tools were developed primarily for evaluation of web content. Given its audio format, implementing traditional forms of referencing can be challenging on a podcast. Accordingly, in the absence of explicit show notes with referencing, we accepted verbal citations that included year and author or the title of an article when discussing evidence in the podcast. Author disclosures and affiliations were also accepted in audio format. We searched for post-publication commentary in either *Apple Podcasts* or on the show's website. We also considered mention of feedback for a specific episode in the following episode as evidence of post-publication commentary.

### Data collection

Two separate authors (HK & LH), a resident doctor and student doctor, respectively, evaluated the five most recent episodes from eligible podcasts using the rMETRIQ and JAMA instruments. We then generated mean scores for each tool. Authors did not confer with one another during the scoring process and were blinded to the scores applied by the other author. Authors were provided with information sheets about each scoring metric and were asked to submit each score using an online survey immediately after observing each episode. Variation between scores > 1 point were adjudicated by an external author not involved in the design of the study or data collection.

Additionally, we collected the following data for each podcast: date of publication, length of podcast, podcast producing body/affiliation, main theme discussed, seniority of the most senior speaker/content reviewer and the seniority of the host or content writer.

### Statistical analysis

We visualized continuous variables by generating histograms, then confirmed their normality visually. We generated means with standard deviations for normally distributed variables, and medians with interquartile range (IQR) for skewed continuous variables. For categorical variables, we generated raw numbers and percentages. We did not adjust for confounding variables when presenting differences between podcast types, as our sample size was inadequately powered for this. We also examined the relationship between individual variables and both rMETRIQ scores as well as JAMA scores by conducting multiple linear regression and multiple logistic regression, respectively. Missing data was sought from Spotify/Apple Podcast databases; missing scoring data was confirmed through triangulated re-appraisals of episodes with three authors. Finally, we calculated an intraclass correlation coefficient to evaluate the agreement between raters using the rMETRIQ and JAMA scales.

## Results

**Figure 1:**
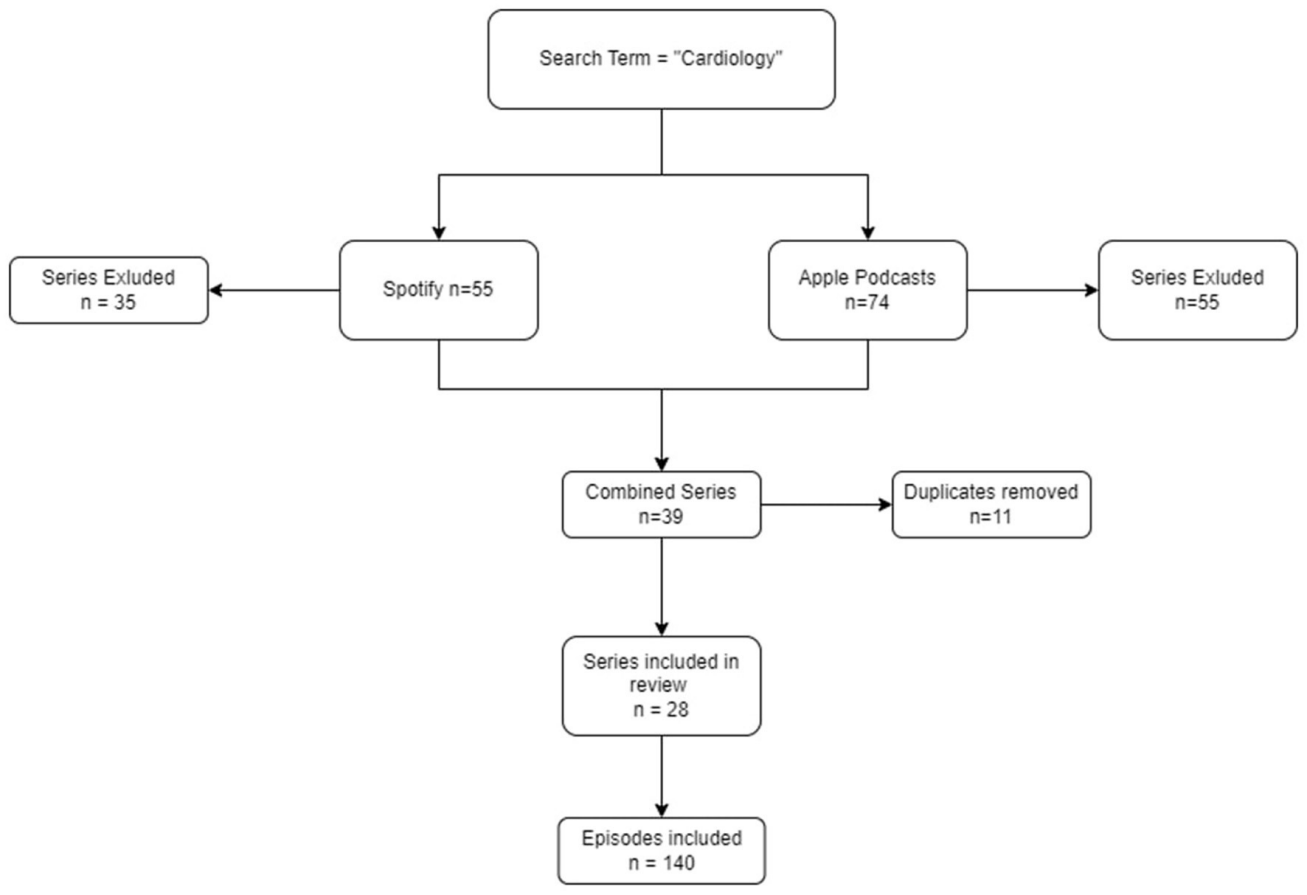
Flow diagram of cardiology podcast series inclusion and exclusion.

This study searched for the top 20 cardiology podcast shows on two respective platforms, Spotify and Apple Podcasts, to evaluate their quality. After executing the search there were 11 duplicate shows that were present on both platforms and one show was excluded after being found not to be clinically focused (Figure 1). Thus, in total there were 28 Cardiology podcast shows which were elligible. The five most recent episodes from each show were retrieved, and so a total of 140 episodes were independently evaluated (Figure 1).

### Podcast characteristics

The median episode length was 20 min. Episode length was highest among podcast episodes affiliated with universities or hospitals (see Table 1). Professors, subspecialty discussants and consultant physicians comprised most podcast hosts (87.14%) and were frequently the most senior discussants on an episode (88.57%). In contrast, doctors in training (registrars, fellows) comprised < 6% of hosts and senior guests on podcast episodes.

**Table 1:** Characteristics of cardiology podcasts.

	**Cardiac society (*n* = 31)**	**Unaffiliated individuals or group (*n*=38)**	**Journal (*n* = 40)**	**Hospital or university (*n* = 31)**	Mean (SD), median (IQR),^*^or number (%)
**Episode length (mins)**	12.7 (14.3)	19.1 (31)	18.6 (11.1)	30.6 (42.2)	20 (19)
**Host**					
Professor or expert	21 (24.1)	15 (17.2)	26 (29.9)	25 (28.7)	87 (62.1)
Consultant	7 (20)	10 (28.6)	13 (37.1)	5 (14.3)	35 (25)
Other	3 (100)	0 (0)	0 (0)	0 (0)	3 (2.1)
Fellow	0 (0)	4 (80)	1 (20)	0 (0)	5 (3.6)
Registrar	0 (0)	4 (100)	0 (0)	0 (0)	4 (2.9)
Not disclosed	0 (0)	5 (83.3)	0 (0)	1 (16.7)	6 (4.3)
**Most senior speaker**					
Professor or expert	25 (24.8)	21 (20.8)	29 (28.7)	26 (25.7)	101 (72.1)
Consultant	5 (21.7)	7 (30.4)	7 (30.4)	4 (17.4)	23 (16.4)
Allied health professional	1 (100)	0 (0)	0 (0)	0 ()	1 (0.71)
Fellow	0 (0)	1 (50)	1 (50)	0 (0)	2 (1.4)
Registrar	0 (0)	4 (57.1)	3 (42.9)	0 (0)	7 (5)
Not disclosed	0 (0)	5 (83.3)	0 (0)	1 (16.7)	6 (4.3)
**rMETRIQ scores**	12.3 (3.2)	13.4 (2.7)	14.6 (2.7)	11.6 (2.5)	13.1 (3)
**Perfect rMETRIQ scores**					
Background information	3 (9.7)	21 (55.3)	27 (67.5)	16 (51.6)	67 (47.9)
Content fits length	24 (77.4)	34 (89.5)	39 (97.5)	22 (71)	119 (85)
Writing and formatting	26 (83.9)	32 (84.2)	36 (90)	23 (74.2)	117 (83.6)
Reference citation	7 (22.6)	4 (10.5)	6 (15)	1 (3.2)	18 (12.9)
Authors and conflicts	8 (25.8)	12 (31.6)	19 (47.5)	4 (12.9)	43 (30.7)
Editorial and peer review	4 (12.9)	7 (18.4)	8 (20)	0 (0)	19 (13.6)
Post-publication commentary	4 (12.9)	6 (15.8)	8 (20)	1 (3.2)	19 (13.6)
**JAMA scores**					
Authorship	31 (100)	37 (97.4)	40 (100)	31 (100)	139 (99.3)
Attribution	18 (58.06)	31 (81.6)	31 (77.5)	9 (29)	89 (63.6)
Currency	31 (100)	36 (94.7)	40 (100)	31 (100)	138 (98.6)
Disclosures	18 (58.06)	25 (65.8)	37 (92.5)	29 (93.6)	109 (77.9)
JAMA perfect score	11 (35.5)	19 (50)	28 (70)	8 (25.8)	66 (47.1)

### rMETRIQ score

The mean rMETRIQ score was 13.1 (SD 3). Compared to episodes affiliated with hospitals or universities, episodes affiliated with journals (b = 3.01, 95% CI 1.7–4.3) or those that were unaffiliated (b = 1.78, 95% CI 0.46–3.1) had higher rMETRIQ scores. The rMETRIQ score difference between hospital/university and society affiliated episodes was non-significant (b = 0.64, 95% CI -0.74–2.03).

Less than half of all episodes provided adequate background information on the topics discussed and guided listeners to other sources of information (Table 1). This proportion was lowest among episodes affiliated with a society (9.7%). Eighty-five percent of episodes had only content that was essential, and 83% of episodes were well written and formatted in a way that optimized learning. Only 12.9% of episodes had references, either in show notes or verbally stated that mapped to specific statements within the podcast, or provided references for statements of fact that may not have been common knowledge. This was limited to a single episode among episodes affiliated with a hospital or university (OR −2.16, 95% CI −4.33 to −0.006). In other words, 87.1% of podcast episodes did not include references.

In more than two-thirds of episodes, the authors of the show were either not identified or conflicts of interest were not declared explicitly. Additionally, 86.4% of podcast episodes did not clearly describe the review process that was applied to the resource. At an equal rate of 86.4%, most podcast episodes failed to expand on their published content with robust post-publication commentary.

We observed no consistent relationship between episode length, seniority of host or seniority of guest speaker with rMETRIQ scores.

### JAMA scores

Less than half of podcast episodes achieved a perfect JAMA score. Authorship was mentioned in 99.3% of episodes, and dates of content posting were provided in 98.6% of episodes. Sixty-three-point 6% of episodes provided clear references or sources, and 77.9% percent of episodes disclosed ownership and any sponsorship, underwriting or commercial funding. Episode length and seniority of speakers demonstrated no consistent relationship with the podcast's JAMA scores.

### JAMA and rMETRIQ correlation

We observed a strong, positive linear relationship between rMETRIQ and JAMA scores, with a 2.5-unit rMETRIQ score increase for every unit increase in JAMA Score (b = 2.56, CI 1.93–3.18). However, 25% of episodes with a perfect JAMA score had rMETRIQ scores less than the mean.

### rMETRIQ + JAMA

We observed a moderate to substantial inter-rater agreement among each of the rMETRIQ scoring items. Despite this agreement, inter-rater agreement on the final score was fair (kappa 0.36, SE 0.02). In contrast, interrater agreement on components of the JAMA score were near perfect (Kappa 0.94–1.00), except for currency (expected equivalent to chance).

## Discussion

The popularity of podcasts as a remote learning tool for clinicians is rising, and this is one of the first studies to systematically assess the quality of English language podcasts published in the field of Cardiology.

We found that most podcast episodes were very well-written and formatted in a way to optimize learning. The podcasts we evaluated also seldom contained unnecessary, redundant, or missing content. We also observed that most episodes were delivered by content experts; in our study, professors, subspecialty discussants or consultant cardiologists led the discussion in the significant majority of episodes. Despite this expertise, we observed no association between the seniority of speakers or hosts and rMETRIQ or JAMA scores. This finding reinforces the work of a modified Delphi consensus study of 44 health profession educators by Lin et al., in which content expertise was not considered a vital quality indicator among medical podcasts (7). Based on these findings, listeners should not be discouraged from streaming content produced by healthcare professionals that are not attending physicians or professors of Cardiology.

Our findings also showed that longer episode duration was not associated with improved podcast quality. Evidence from a recent scoping review of podcasts in medical education by Kelly et al. and data from Cosimini et al. suggests that residents and medical students consistently prefer podcast episodes between 5 and 15 min, with longer lengths serving as a barrier to uptake for listeners (6, 14). Taken together, these findings suggest that cardiology podcast producers can aim for shorter podcast episode lengths and increase audience accessibility, without sacrificing quality.

Additionally, our study also identified several important areas where popular cardiology podcasts could improve; only one out of 140 episodes scored a perfect rMETRIQ score whilst < $\frac{1}{2}$ of all episodes scored a perfect JAMA score. Less than half of podcast episodes provided sufficient background information to situate the listener *and* directed listeners to other valuable resources to the topic. Situating a listener in the broader context of a discussion is important to improve understanding and maximize engagement (12, 15). References to alternative, related material also enhances comprehension, particularly among listeners without expertise in the subject matter (4, 16). Providing essential background information may facilitate podcast material uptake, particularly among novice listeners.

Referencing was also limited in the episodes we evaluated. Some form of referencing was present in under two thirds of episodes (JAMA Attribution = 63.6%), but using the rMETRIQ tool, we found that only 12.9% of podcast episodes provided references that clearly mapped to specific statements made within the episode and provided references for statements of fact that were not common knowledge. Compared to traditional modes of medical education, the podcast format is unique in the way it facilitates both instant dissemination of information and discussion. However, statements of opinion can unintentionally be presented as statements of fact (16). Clear referencing of statements of fact not considered common knowledge is necessary to prevent listeners from conflating the two, especially among topics under active debate within cardiology.

Our study also found that evidence of peer review and post-publication commentary was limited. Only 13.6% of episodes we evaluated provided a review process *and* evidence of its application to the specific episode. Subjecting scientific discussion to peer-review is vital to identify factual errors, provide alternate points of view and identify any inherent biases within the discussion (16). Additionally, only 13.6% of episodes also provided evidence of a robust discussion of the episode's content that expanded upon the content of the episode. These findings may be due to the logistical challenges of facilitating robust discussion from listeners on a podcast. However, given that most podcast episodes provided avenues for listeners to leave feedback, discussion and exploration of this feedback may reinforce the resource's trustworthiness and enhance uptake (7, 17).

Finally, statements of authorship were almost universally present (JAMA Authorship, 99.3%), and statements regarding commercial funding were disclosed in over three quarters of episodes (JAMA Disclosures = 77%). However, statements regarding the presence or absence of any conflict of interest *related to hosts or guests* were present in less than a third of podcast episodes as observed using the rMETRIQ tool (30.7%). This is an important finding, as we found that most podcasts are hosted by, or have guests that are, content experts. Content experts may be affiliated with industry (16, 18) and mention of these affiliations is important to contextualize unintentional bias inherent in the presented points of view.

Our *a priori* hypothesis was that cardiac societies would produce podcasts with higher rMETRIQ and JAMA Scores. Our findings do not support this hypothesis. Cardiac societies frequently produce peer-reviewed, evidence-based guidelines that often determine standards of care in cardiology (19). We hypothesized that this experience would translate into the production of well-produced, reliable and unbiased medical education content designed to promote evidence-based therapy among podcast listeners. However, compared to episodes from journals or episodes that were unaffiliated, cardiac society episodes scored lower rMETRIQ scores on average (vs. Journal β: −2.70, 95% CI: −3.99 to −1.42; vs. Unaffiliated β: −1.91, 95% CI: – 3.24 to −0.58;). This was mainly driven by differences in background information, mention of conflicts of interest, evidence of peer review and demonstration of post-publication commentary. We observed no other association between episode length, seniority of host or seniority of guest with our outcome measures (data not presented).

Our study has also highlighted the strengths and weaknesses of the rMETRIQ and JAMA tools in evaluation of podcasts. Both tools were correlated in our study, with a 2.5-unit rMETRIQ score increase for every unit increase in JAMA Score (b = 2.56, CI 1.93–3.18). However, both tools demonstrated important limitations. For instance, the JAMA tool failed to interrogate important aspects of podcast quality evaluated by rMETRIQ, such as background information, appropriateness of content for length, writing and formatting, peer-review, and post-publication commentary. Even in areas of overlap, the JAMA score was generous; in our study, 25% of episodes with perfect JAMA scores had rMETRIQ scores less than the mean (13.1). We also found the currency metric of the JAMA score to be redundant, as podcast providers automatically display the dates of publication for a given episode. Despite these limitations, we found that the JAMA score is an easy, reliable (Table 2), four-step tool that a listener can rapidly apply to gauge the quality of an episode. Although the rMETRIQ tool interrogates important aspects of a podcast, it can be time consuming, and as we have shown, has lower inter-rater reliability (Table 3). In fact, our rMETRIQ scores represented optimistic scoring; where scores did not match, a third author adjudicated the final rMETRIQ score for each episode. In 10% of cases, the final score was different to the scores generated by each of the first two authors, and in all these cases, the final score was higher. Our results suggest that neither the rMETRIQ or JAMA rating tool is perfect, and a gap in the literature exists for the development of a validated medical podcast rating tool that is reliable and conveniently applied.

**Table 2:** Kappa statistic of inter-rater agreement between individual JAMA items and total score.

	**Agreement (%)**	**Expected (%)**	**Kappa**	**Std. Err**.
Authorship	100	98	1.00	0.08
Attribution	97	53	0.94	0.08
Currency	97	97	0.00	0.08
Disclosures	98	64	0.94	0.08
**Total**	97	42	0.95	0.06

**Table 3:** Kappa statistic of inter-rater agreement between individual rMETRIQ items and total score.

**rMETRIQ item**	**Agreement (%)**	**Expected (%)**	**Kappa**	**Std. Err**.
Item 1	73	40	0.55	0.06
Item 2	91	69	0.72	0.08
Item 3	87	65	0.63	0.08
Item 4	82	27	0.76	0.05
Item 5	78	41	0.63	0.06
Item 6	88	61	0.69	0.06
Item 7	86	29	0.81	0.05
Overall	42	9	0.36	0.02

### Strengths and limitations

Our study has some limitations. Firstly, the podcasts evaluated were limited to English language podcasts published on *Spotify* and *Apple Podcasts*. An important limitation of this approach is that the *Spotify* and *Apple Podcasts* search algorithm return podcasts in order of relevance according to the user's profile to reproduce typical user behavior (20, 21). This means that at the time of search, behavioral data and engagement data influenced the cardiology shows displayed for any given search term, by any given consumer. To mitigate this issue, we replicated the search among two independent investigators and removed duplicates. We chose this search strategy to emulate real-world practices in podcast consumption but recognize that this may have yielded fewer podcasts for analysis. A further limitation is that the rMETRIQ and JAMA tools were generated for critical appraisal of written publications; in the absence of existing tools to evaluate podcasts, we opted to use these scoring systems given their extensive use in the literature with audio-visual and scripted formats (22–24) formats. Finally, a lack of publicly available data on podcast downloads prevented us from analyzing how podcast quality relates to listenership.

Despite these limitations, there are important strengths to our study. This study is among the first to apply critical appraisal using validated instruments to medical content in cardiology podcasts. In doing so, we provide a clear framework through which listeners can evaluate the quality of the content they consume prior to implementing this information in clinical practice. Our study also engaged authors to conduct analyses independently to avoid inadvertent bias in scoring, with engagement of a third, external author to adjudicate inconsistencies in scoring. This was done to maximize reliability and reproducibility among scoring estimates. Finally, our methodology employed a pragmatic, real-world approach to searching for and consuming medical content, designed to emulate the experience of busy clinicians and students searching for medical education.

## Conclusion

Cardiology podcasts are becoming increasingly popular and have potential to influence clinical decisions worldwide. Contrary to our hypothesis, the quality of cardiology podcasts varies widely and those produced by professional bodies did not necessarily achieve higher scores on tools designed to assess online medical education. Producers should strive to increase transparency of the review process and the evidence-base driving their discussions. Future research in this area should focus on developing streamlined criteria for evaluating the quality of podcasts. Ultimately, cardiology podcasts remain a valuable remote learning tool for clinicians. Our study has identified important deficits in their evaluation and provide a framework for future efforts to ensure their reliability, relevance and transparency.

## Data availability statement

The raw data supporting the conclusions of this article will be made available by the authors, without undue reservation.

## Author contributions

HK: Conceptualization, Investigation, Methodology, Writing – original draft, Writing – review & editing. LH: Conceptualization, Investigation, Writing – review & editing. SB: Conceptualization, Methodology, Resources, Supervision, Writing – review & editing. WM: Data curation, Formal analysis, Writing – original draft, Writing – review & editing. AZ: Data curation, Writing – review & editing. FS: Writing – review & editing. JK: Writing – review & editing. AG: Conceptualization, Methodology, Writing – review & editing. PP: Supervision, Writing – review & editing. PK: Writing – review & editing.
