# Supplementary figures and images for: Listen to your heart: a critical analysis of popular cardiology podcasts
Source: Front Med (Lausanne). 2024 Jul 15;11:1278449. doi: 10.3389/fmed.2024.1278449 (PMC11299239; doi:10.3389/fmed.2024.1278449)

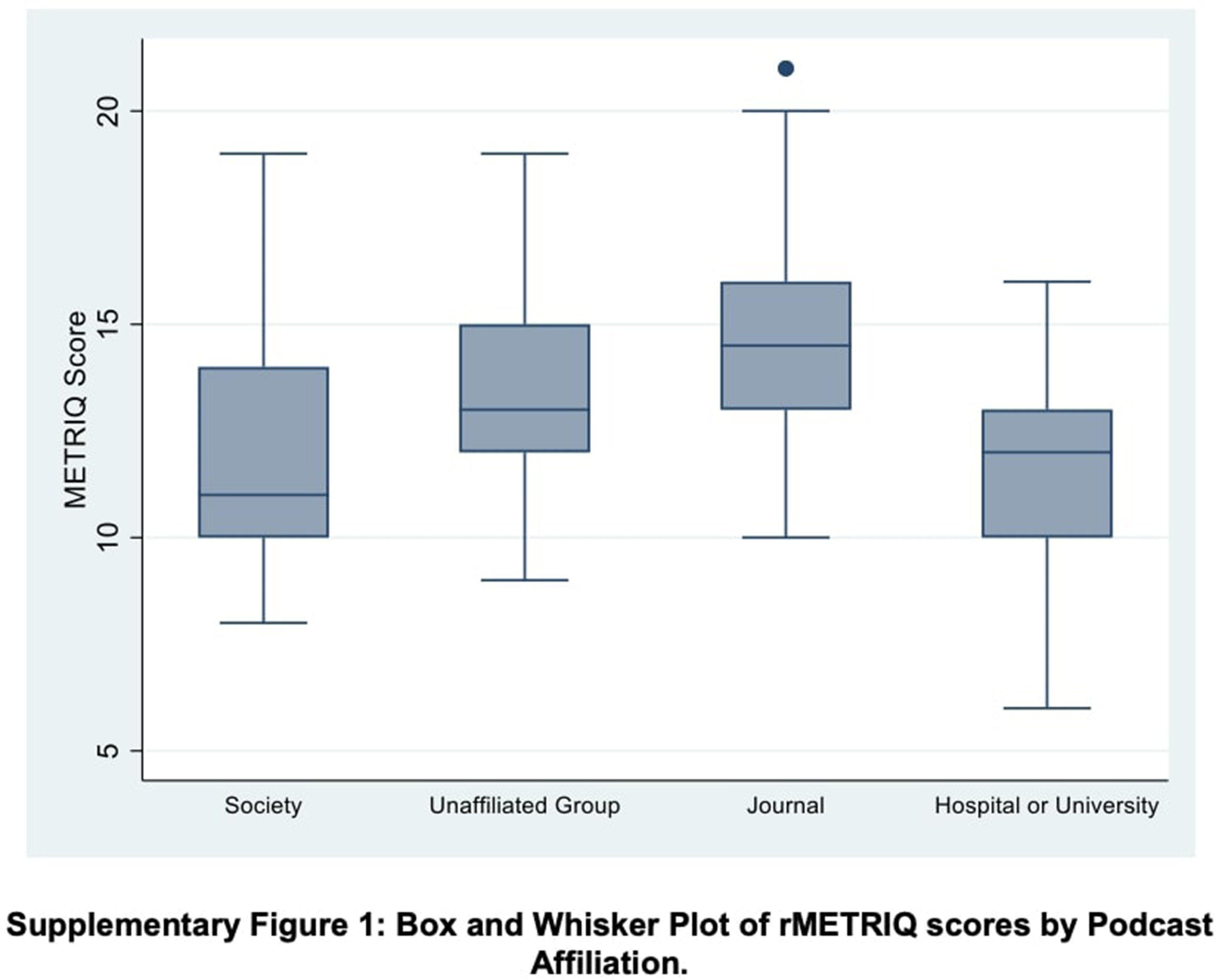

Supplement: Supplementary file 1 [file Image_1.JPEG]

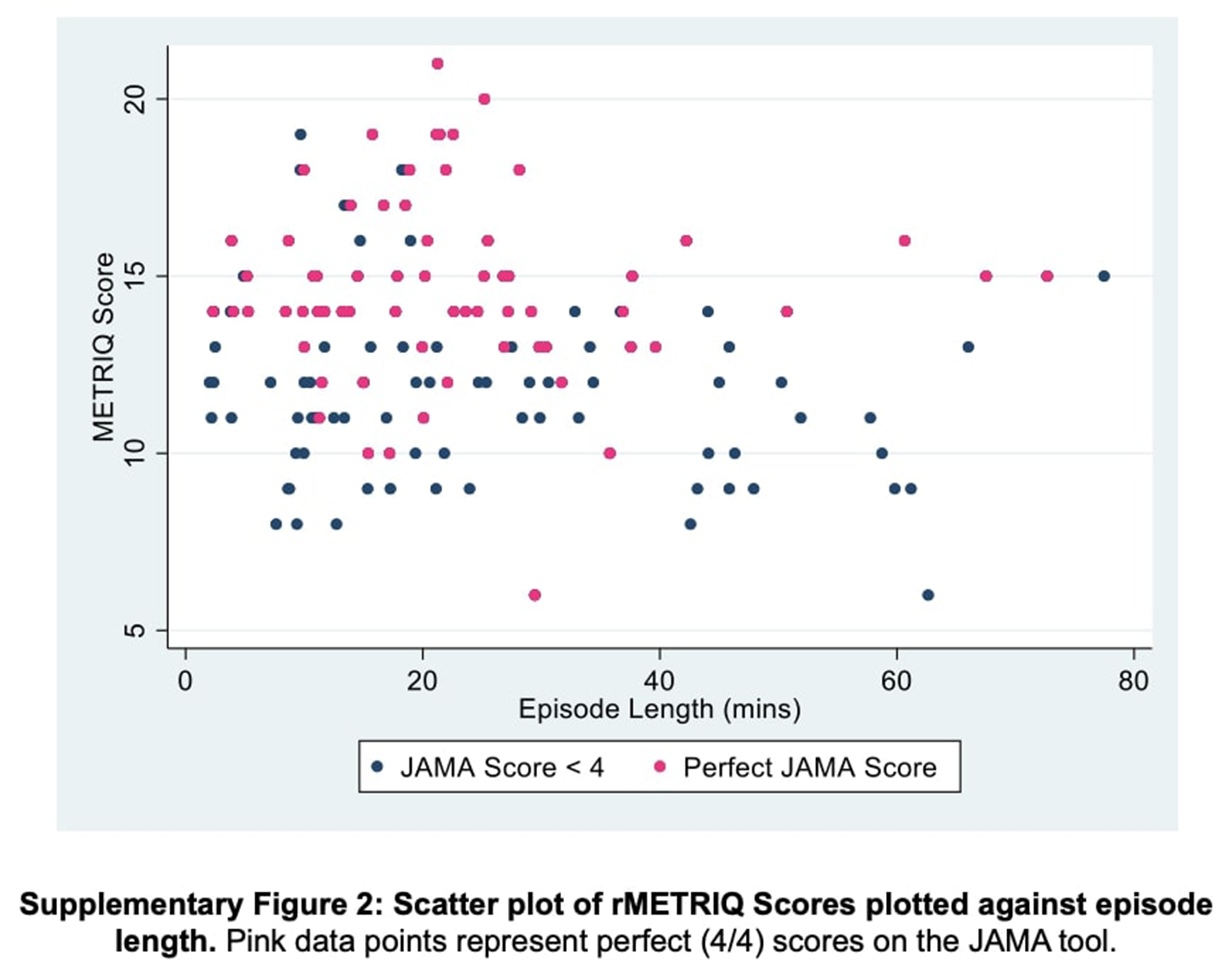

Supplement: Supplementary file 2 [file Image_2.JPEG]

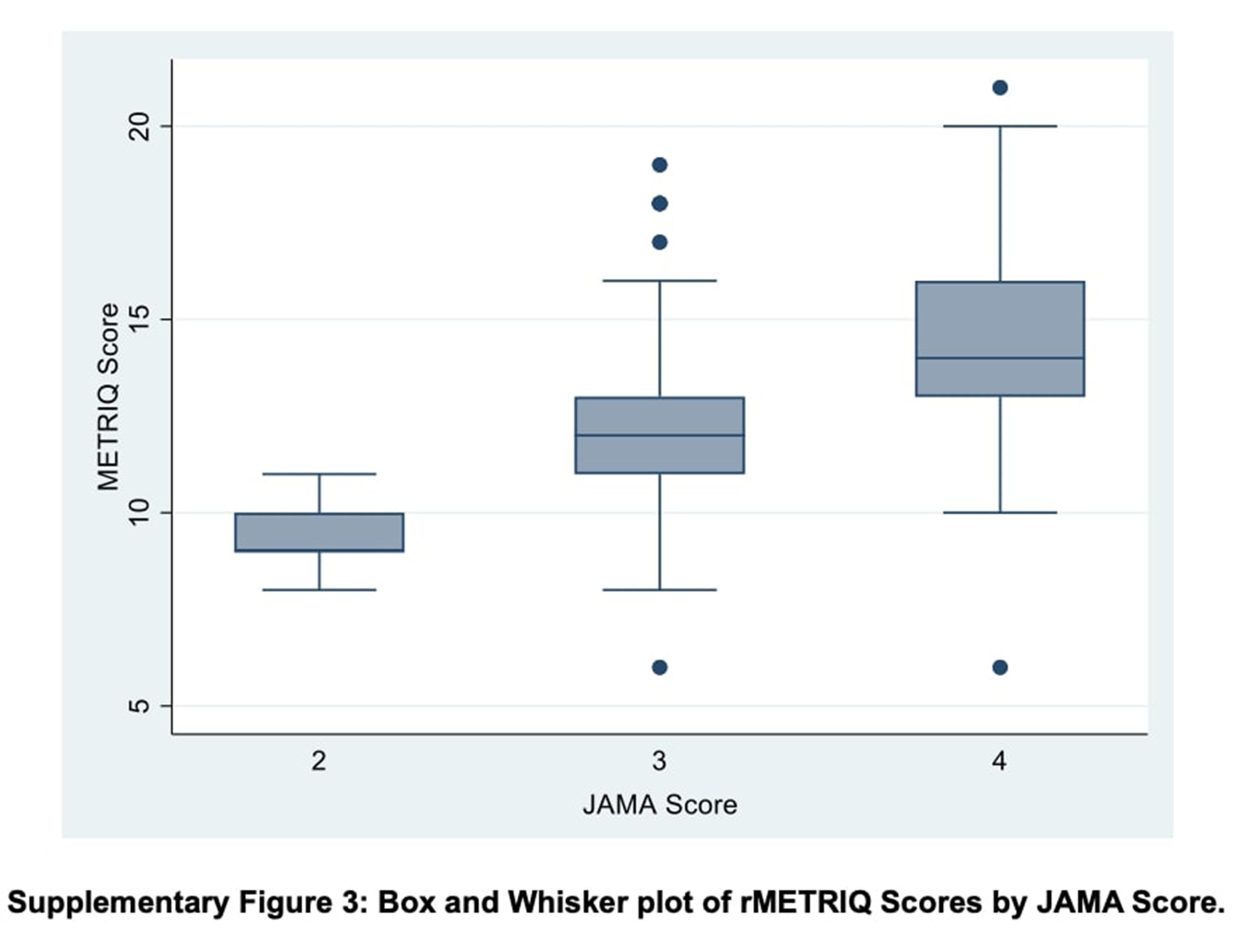

Supplement: Supplementary file 3 [file Image_3.JPEG]
